# Sirenomelia: Adding Something New

**Published:** 2012-01-01

**Authors:** Bilal Mirza

**Affiliations:** Department of Paediatric Surgery, The Children's Hospital and the Institute of Child Health Lahore, Pakistan

**Keywords:** Sirenomelia, Mermaid syndrome, Variant, Twins

## Abstract

A case of sirenomelia is being reported who was born in a twin pregnancy and showed different anatomy of lower torso.

## INTRODUCTION

Sirenomelia is a very rare birth anomaly. It is classified in three different anatomic types based on clinical and radiological findings [1-3]. Herein we are reporting a case of sirenomelia who showed different anatomy of lower limb.

## CASE REPORT

A few hours old newborn was brought to our unit with fused lower torso. The pregnancy outcome was twins; the other female baby was alright. It was a full term pregnancy (primigravida) and a product of non-consanguineous marriage. There was no history of any teratogenic drug use and maternal diabetes during pregnancy. On examination the weight of baby was 2kg; there was a single lower limb with two feet (most distally). The plantar and dorsal aspects of foot were reversed (Fig. 1). There were absent scrotum/gonads, and anal opening. A phallus like skin tissue was present without any urethral opening in it. On palpation one bone was palpable in thigh and two equal bones in the leg. X ray of the baby showed one femur and two tibiae (Fig. 2). Ultrasound of the baby showed bilateral renal agenesis and bladder/gonads were not visualized. The other twin was quite normal. The parents were counseled about the outcome. They took the baby home where the baby expired on 5th day of life.

**Figure:**
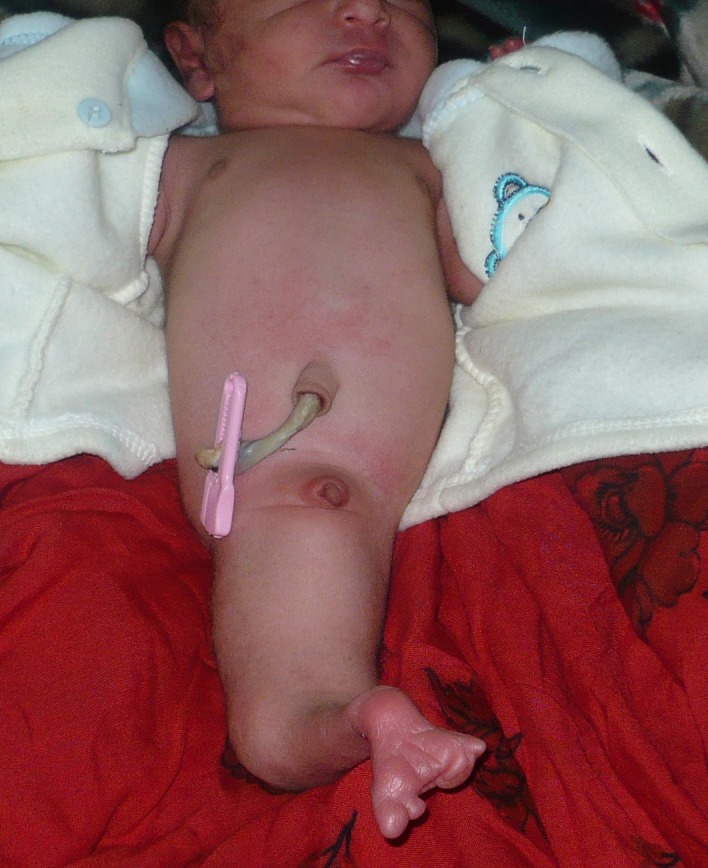
Figure 1: Sirenomelia

**Figure:**
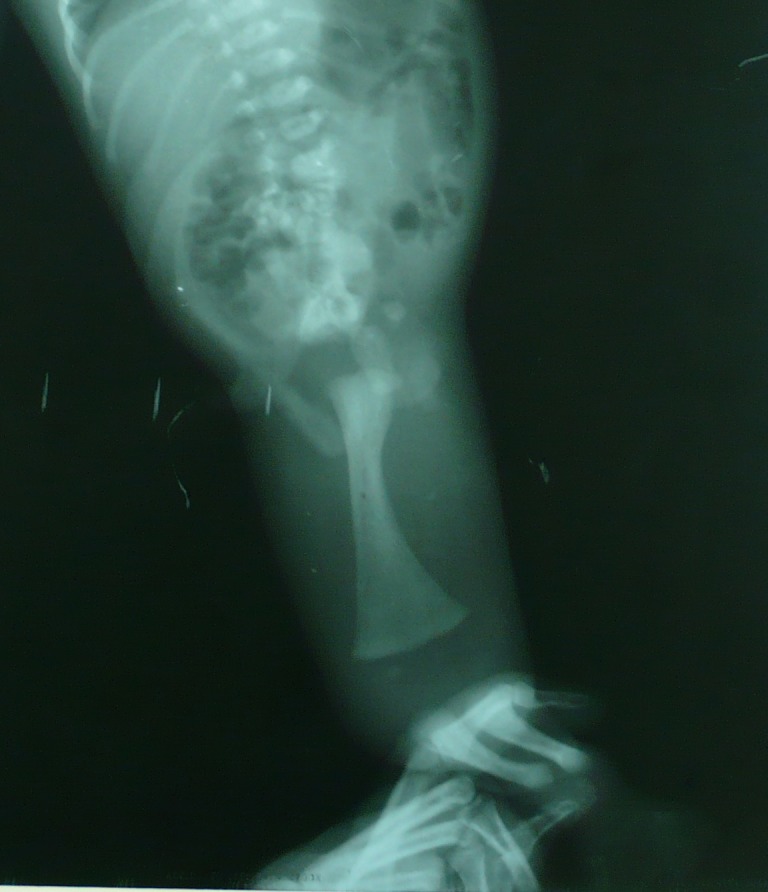
Figure 2: X ray showing one femur and two tibiae

## DISCUSSION

The sirenomelia has been classified into Simpus apus (no feet, one tibia, one femur), Simpus unipus (one foot, two tibia, two fibula, two femur), and Simpus dipus (two feet, two fused legs) flipper like popularly known as mermaid. In our case the patient had two fused feet and one femur and two tibial bones. This variety does not fit into any of the mentioned varieties. This variety may be called as Simpus dipus variant. The other peculiar finding in our case was twin pregnancy which is very rare. More interestingly, the other twin was quite normal thus excluding environmental factors, teratogenic drugs, and maternal diabetes from the etiological factors as both of the twins would had been equally exposed to predisposing factors. It could be a genetic aberration however karyotyping in these patients is always reported as normal [1-3].

## Footnotes

**Source of Support:** Nil

**Conflict of Interest:** None declared

